# Authors’ Response: Comment on: “Transversus Thoracic Muscle Plane Block for Attenuating the Haemodynamic Response to Median Sternotomy”

**DOI:** 10.4274/TJAR.2023.231423

**Published:** 2023-08-18

**Authors:** Ashish Walian, Rohan Magoon, Iti Shri, Ramesh Chand Kashav

**Affiliations:** 1 Department of Cardiac Anaesthesia, Atal Bihari Vajpayee Institute of Medical Sciences (ABVIMS) and Dr. Ram Manohar Lohia Hospital, New Delhi, India

**Dear Editor,**

We thank Sethuraman^1^ for an interested reading of our case series^2^ and would wish to respond to the points raised.

Firstly, the correspondence discusses the concerns about referencing in our manuscript.^1,2^ Without denying the significance of refraining from citing retracted literature, it remains to be put forth that these articles were cited only from a technical purview of the subject and were in no form capable of affecting our findings.^3^ Having said that, we do regret the inability to replace the former with alternative articles, in the later stages of the publication process. Meanwhile, the retraction of the cited literature stands notified to the readers in the bibliography, we simultaneously take the responsibility for having presented our clinical experience with utmost sincerity and again appreciate Sethuraman^1^ for pointing out an important shortcoming in our drafting endeavors.^2^

Secondly, Sethuraman^1^ questions our choice of citing the Taketa et al.^4^ case series which features the use of erector spinae plane block (ESPB). It is understandable that on a superficial perusal, an ESPB paper might not appear to have anything to do with a case series on transversus thoracic muscle plane block (TTPB). Nonetheless, Taketa et al.^4^ categorically emphasize the dermatomal role of anterior cutaneous branches of the intercostal nerves in the context of parasternal analgesia. The blockade of these branches becomes pivotal with regards to a sternotomy incision and hence, the citation in a case series proposing to achieve the same with TTPB.^2^

Lastly, being mindful of the multifactorial etiology of acute post-cardiac surgical pain, the findings of our case series specifically focusing on the hemodynamic response to median sternotomy has a distinct meaning in our opinion.^2,5^ As far as the continued reliance on hemodynamic surrogates in analgesic research is concerned, the need for reliable alternate intraoperative nociception monitoring is certainly making for some ardent debates amongst the perioperative fraternity.^5^
